# Epidemiological Characteristics and Spatiotemporal Analysis of Mumps from 2004 to 2018 in Chongqing, China

**DOI:** 10.3390/ijerph16173052

**Published:** 2019-08-22

**Authors:** Hua Zhu, Han Zhao, Rong Ou, Haiyan Xiang, Ling Hu, Dan Jing, Manoj Sharma, Mengliang Ye

**Affiliations:** 1 Department of Epidemiology and Health Statistics, School of Public Health and Management, Chongqing Medical University, Chongqing 400016, China; 2 Chongqing Municipal Center for Disease Control and Prevention, Chongqing 400042, China; 3 Department of Medical Informatics Library, Chongqing Medical University, Chongqing 400016, China; 4 Department of Behavioral and Environmental Health, Jackson State University, Jackson, MS 39213, USA

**Keywords:** mumps, epidemiology, spatiotemporal analysis, cluster

## Abstract

Mumps vaccines have been widely used in recent years, but frequent mumps outbreaks and re-emergence around the world have not stopped. Mumps still remains a serious public health problem with a high incidence in China. The status of mumps epidemics in Chongqing, the largest city in China, is still unclear. This study aimed to investigate the epidemiological and spatiotemporal characteristics of mumps and to provide a scientific basis for formulating effective strategies for its prevention and control. Surveillance data of mumps in Chongqing from January 2004 to December 2018 were collected from the National Notifiable Diseases Reporting Information System. A descriptive analysis was conducted to understand the epidemiological characteristics. Hot spots and spatiotemporal patterns were identified by performing a spatial autocorrelation analysis, a purely spatial scan, and a spatiotemporal scan at the county level based on geographic information systems. A total of 895,429 mumps cases were reported in Chongqing, with an annual average incidence of 36.34 per 100,000. The yearly incidence of mumps decreased markedly from 2004 to 2007, increased sharply from 2007 to 2011, and then tapered with a two-year cyclical peak after 2011. The onset of mumps showed an obvious bimodal seasonal distribution, with a higher peak of mumps observed from April to July of each year. Children aged 5–9 years old, males, and students were the prime high-risk groups. The spatial distribution of mumps did not exhibit significant global autocorrelation in most years, but local indicators of spatial autocorrelation and scan statistics detected high-incidence clusters which were mainly located in the midwestern, western, northeastern, and southwestern parts of Chongqing. The aggregation time frame detected by the purely temporal scan was between March 2009 and July 2013. The incidence of mumps in Chongqing from 2004 to 2018 featured significant spatial heterogeneity and spatiotemporal clustering. The findings of this study might assist public health agencies to develop real-time space monitoring, especially in the clustering regions and at peak periods; to improve immunization strategies for long-term prevention; and to deploy health resources reasonably.

## 1. Introduction

Mumps, an acute respiratory infectious disease caused by the mumps virus, is transmitted to a person primarily through contact, fomites, and droplets [1]. The main clinical manifestation of mumps is unilateral or bilateral parotid gland swelling, accompanied by pain and fever [2]. Although its common symptoms seem mild, mumps can cause serious complications such as permanent deafness, encephalitis, pancreatitis, oophoritis, and orchitis, which may result in disability or death and bring a heavy economic burden to families and societies [3,4]. This disease is highly contagious and often occurs in childhood [5]. Since the mumps vaccine was approved for use in 1967, mumps has become a vaccine-preventable infectious disease, and the incidence has rapidly and substantially reduced. By the end of 2017, the mumps vaccine had been introduced worldwide in 122 countries [6]. The specific effect of vaccination is related to factors such as age, coverage, and inoculation times. However, the recovery and outbreaks of mumps have not stopped, even in some developed countries with higher immunization rates, such as the United States [7], France [8], Britain [9], and Australia [10], which has once again aroused concern and attention. Increasing evidence has suggested that vaccine-induced immunity impairment is not sufficient to fully prevent mumps outbreaks and that an advanced strategy of immunotherapy is required—three doses of vaccine rather than two [11,12,13,14]. The number of mumps cases in China has declined in recent years, but it ranked first among all countries in 2017 [15]. From 2004 to 2017, the total number of mumps cases in China reached 402,520, accounting for 51.41% of the total global cases [15]. In 2008, China added the mumps-containing vaccine (MuCV) to its national immunization program, providing children aged 18–24 months one dose of measles–mumps–rubella (MMR) vaccine free of charge. However, only a few economically developed areas implemented the two doses of the vaccine. Therefore, the level of immunization in China as a developing country lags behind many developed countries, which have widely implemented the second dose of the vaccine schedule. Mumps is still a significant public health concern in China and thus deserves to be explored and studied.

Geographic information systems (GIS) are widely applied in the field of public health [16] and are an effective tool to describe the spatiotemporal distribution, evolution, and aggregation of a disease as well as visualize morbidity and clustering regions on a map [17,18]. Researchers have generally applied spatial autocorrelation and space–time scan techniques to explore the spatial and temporal characteristics of various communicable diseases [19,20,21,22,23], which has contributed to the adoption of improved preventive and control measures for regions and periods with different risk levels. Previous studies on mumps in terms of spatial and temporal analyses showed spatial dependence and heterogeneity in several regions of China, such as Shandong [24], Guangxi [25], and Gansu [26]. Chongqing, a central-government-controlled municipality located in the southwest of China and the upper reaches of the Yangtze River, is the largest and most populous city in China. To the best of our knowledge, no study has been carried out on the epidemiological and spatiotemporal features of mumps in Chongqing, which has remained a knowledge gap.

Therefore, in this study, we conducted a descriptive and geographical analysis at the county level, based on the surveillance data of mumps in Chongqing from 2004 to 2018, to identify the epidemiological characteristics and discover the spatiotemporal pattern of mumps.

## 2. Materials and Methods

### 2.1. Study Area

**Figure 1:**
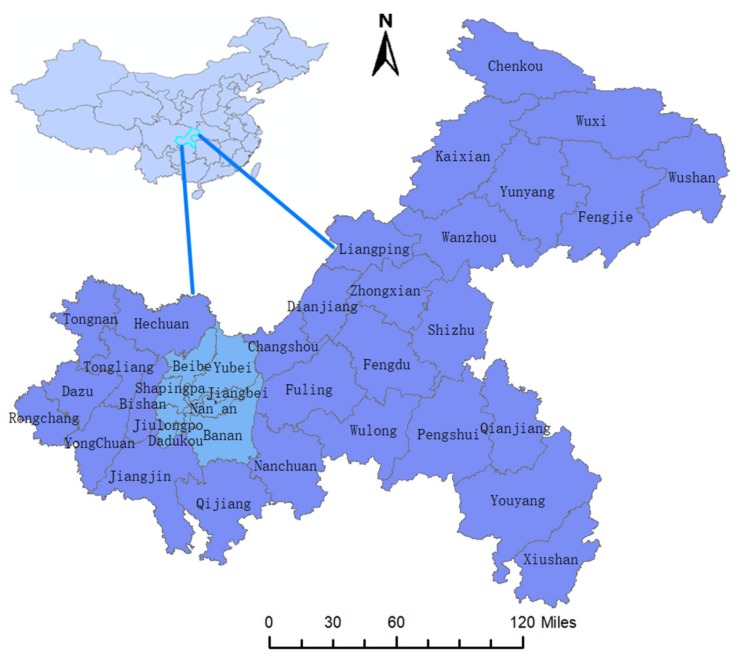
The geographical location and administrative division of Chongqing at the county level in China. The blue part represents the nine main urban districts.

Chongqing (28°10′–32°13′ N, 105°11′–110°11′ E), the largest city and economic center of southwest China, covers a land area of 824,000 km^2^ with 38 districts and counties and had an inhabitant population of about 31.01 million by the end of 2018 (Figure 1). Chongqing is mainly dominated by mountains (76%) and hills and belongs to a subtropical humid monsoon climate zone with a moist climate, ample precipitation, and four distinct seasons. The weather is relatively moderate, with an annual average temperature of 16–18 °C. The main urban area of Chongqing consists of nine districts, namely, Yuzhong, Jiangbei, Nanan, Jiulongpo, Shapingba, Dadukou, Beibei, Yubei, and Banan, which are pinpointed in Figure 1.

### 2.2. Data Collection and Management

The National Notifiable Diseases Reporting Information System (NNDRIS), established in 2004, covers all kinds of medical and health institutions at all levels throughout the country [27]. Patients with mumps as a Class C infectious disease are diagnosed by professional doctors on the basis of the clinical manifestations and laboratory examination results according to diagnostic criteria for mumps promulgated by China’s Ministry of Health. According to the regulations of the Health Administrative Department under the State Council, doctors must fill out infectious disease report cards, which are collected by epidemic report management personnel of hospitals or public health institutions and delivered to the NNDRIS within 24 h. Surveillance data of mumps from January 2004 to December 2018 in various districts and counties of Chongqing were obtained from NNDIRS, including sociodemographic characteristics, date of onset, address, and so on. The occupations of patients with mumps were classified according to the occupational classifications set by the standardized form on the infectious disease report card of the People’s Republic of China [28]. The incidence of mumps was calculated by dividing the number of new reported mumps cases by the population and expressed as cases per 100,000 persons. Population data were provided by the Chongqing Center for Disease Control and Prevention. The vector maps of China and Chongqing came from the National Basic Geographic Information System.

### 2.3. Spatial Autocorrelation Analysis

In this study, the purpose of the spatial autocorrelation analysis was to probe the correlation from the spatial domain and search for potential cluster regions, which mainly included global and local spatial autocorrelation analyses. The global spatial autocorrelation with the indicator of global Moran’s *I* statistic reflected the spatial dependence of mumps incidence in a global area. The Moran’s *I* ranged from −1 to 1. An *I* > 0 and an *I* < 0 with statistical significance represented positive and negative spatial autocorrelation, respectively. An *I* = 0 indicated no spatial autocorrelation, which meant that the mumps was randomly distributed throughout Chongqing. *p*-values (*p* < 0.05) and Z-scores (|Z| < 1.96) were utilized to determine the statistical significance of Moran’s *I*. Specific cluster patterns and regions were detected by local spatial autocorrelation, which was expressed by local indicators of spatial autocorrelation (LISA) [29,30]. Four spatial patterns could be seen intuitively in LISA maps: high–high (high-incidence regions surrounded by high-incidence regions); high–low (high-incidence regions surrounded by low-incidence regions); low–low (low-incidence regions surrounded by low-incidence regions); and low–high (low incidence surrounded by high incidence).

### 2.4. Scan Statistics

SaTScan software was used to perform retrospective purely temporal, purely spatial, and space–time scan analyses, with a discrete Poisson model only for high-incidence areas. The basic idea of scan analysis is to set up a scanning window that can move in time and/or space, and the size and location of the window change dynamically [31]. The windows of purely temporal, purely spatial, and space–time scans are defined by an interval, either circular or elliptical, corresponding to the geographic areas and a cylinder with a circular or elliptical base, respectively. The bottom and height of the cylinder correspond to the geographical area and the length of time, respectively. In the scan statistics, the null hypothesis was that the relative risk (*RR*) of the disease was the same within the scan window compared to the outside, which suggested that the spatial and/or temporal distribution of mumps was completely random, while the alternative hypothesis was that the risk of disease within the window was higher than the outside. In this study, the maximum spatial cluster size was specified as 20% of the population at risk in the spatial window, and the maximum temporal cluster size as 50% of the study period in the time window. Then, the log likelihood ratio (*LLR*) was calculated on the basis of the actual and theoretical number of cases inside and outside the window, and the *p*-values were computed through a Monte Carlo hypothesis test. The cluster with the largest *LLR* value was considered as the most likely cluster. In the purely spatial and space–time analyses, SaTScan also identified secondary clusters in the dataset, in addition to the most likely cluster, and ordered them according to their likelihood ratio test statistic. The number of Monte Carlo simulations was set to the default value of 999.

### 2.5. Statistical Software

In this study, a descriptive statistical method was used to investigate the population distribution and seasonal characteristics of mumps cases. Spatial autocorrelation analysis was conducted using OpenGeoda software version 1.2.0 [32]. The three scan methods above were performed using SaTScan software version 9.5 (Martin Kulldorff, National Cancer Institute, Bethesda, MD, USA). Geographical distribution, local clustering regions of annual incidence, and scanning results were mapped and visualized by ArcGIS software version 10.2 (ESRI, Redlands, CA, USA). All results were considered statistically significant when the *p*-value for both sides was less than 0.05.

## 3. Results

### 3.1. Epidemiological Characteristics

**Figure 2:**
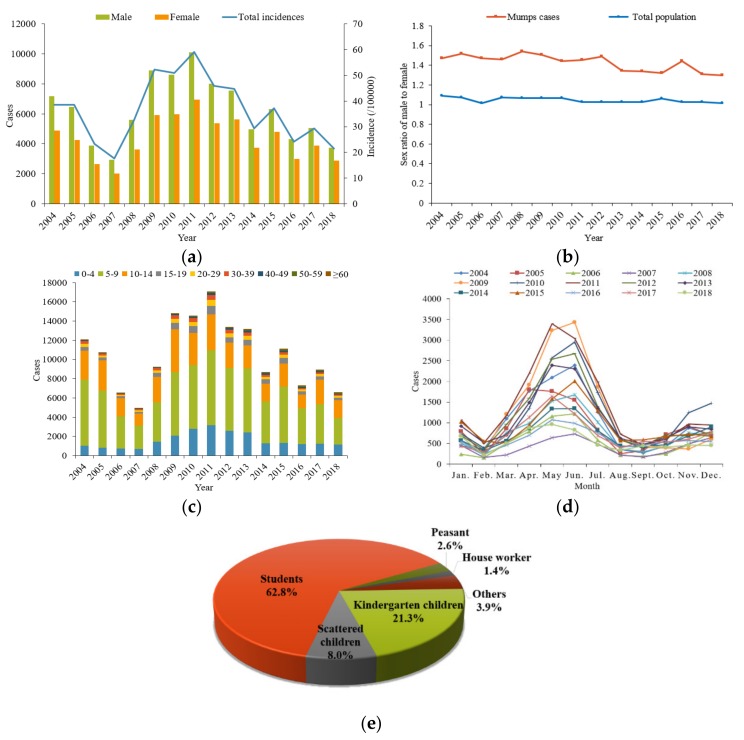
Epidemiological characteristics of mumps in Chongqing from 2004 to 2018. (**a**) The annual case number of mumps across different genders and the annual incidence of total mumps cases. (**b**) The annual sex ratio of male to female for mumps cases and total population. (**c**) The aged distribution of mumps cases. (**d**) The monthly distribution of mumps cases. (**e**) The occupational distribution of mumps cases.

A total of 895,429 mumps cases were reported in Chongqing from 2004 to 2018, with an annual average reported incidence of 36.34 per 100,000 (ranging from 17.55 per 100,000 in 2007 to 59.14 per 100,000 in 2011). The changing tendency of mumps occurrence in the whole of Chongqing was divided into three stages: the incidence of mumps decreased from 2004 to the lowest level in 2007; then, it increased to the peak in 2011; finally, from 2011 to 2018, the overall epidemic trend was downward with a two-year periodic peak. There were 93,655 male cases and 65,526 female cases reported, respectively. The average sex ratio of male to female was 1.42:1, higher than that of Chongqing’s total population. With regard to the age distribution, cases of patients aged 5–9 years ranked first, accounting for 48.8% of the total cases, followed by patients aged 10–14 (24.2%) and patients aged 0–4 (15.1%). In terms of the occupation classification, students were the leading group (62.8%), followed by kindergarten children (21.3%) and scattered children (children who have not reached the age of 3 years to attend kindergarten or are taken care of by their family members) (8.0%). The prevalence of mumps had obvious seasonality. The primary peak period of mumps occurred from April to July (57.5%), while the second peak occurred from November to January in the next year (20.0%). All of the results above are shown in Figure 2.

### 3.2. Incidence Maps

**Figure 3:**
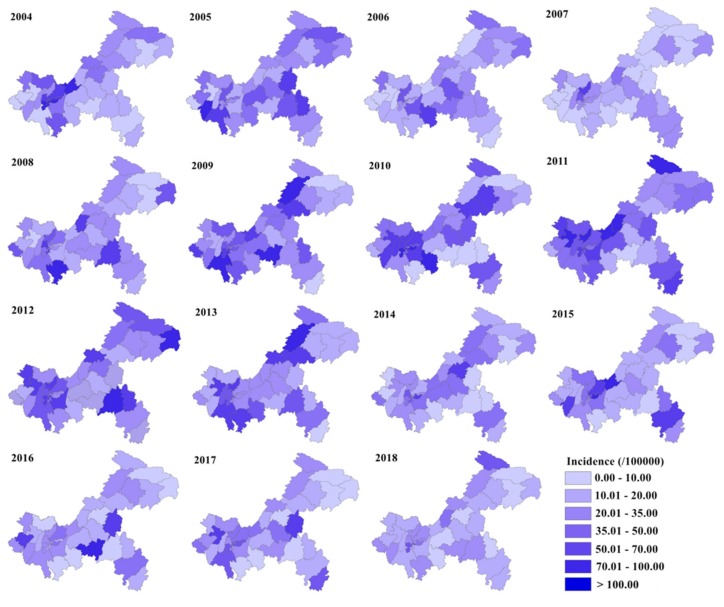
Annual incidence maps of mumps in Chongqing from 2004 to 2018.

Annual incidence maps of mumps infection across the years 2004–2018 (Figure 3) indicated that the location of high-incidence counties (districts) in Chongqing changed but was mainly in the west and northeast. The top five districts in terms of annual incidence were Jiulongpo (61.92 per 100,000), Beibei (61.57 per 100,000), Nan’an (59.98 per 100,000), Shapingba (58.58 per 100,000), and Dadukou (51.39 per 100,000), all located in the main urban area of Chongqing city. Mumps occurrence was more prevalent and the geographical distribution more heterogeneous during the period of 2009–2013. Nevertheless, over the last few years, the mumps epidemic showed a decreasing trend, accompanied by smaller regional variation.

### 3.3. Spatial Autocorrelation Analysis

**Figure 4:**
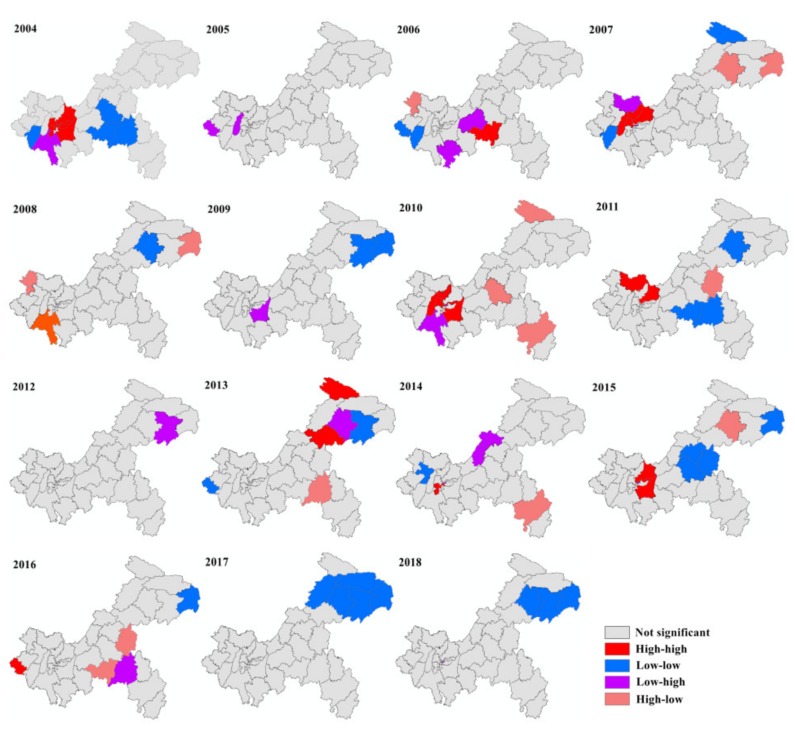
Yearly local spatial autocorrelation of mumps in Chongqing from 2004 to 2018.

The results of the global autocorrelation analysis in Chongqing in 2004–2018 are listed in Table 1. Significant positive global correlations existed in 2004, 2007, and 2015, and the distribution of mumps demonstrated no spatial correlation over the entire region in other years. According to the yearly LISA cluster maps of mumps, the local spatial autocorrelation analysis detected 16 high–high, 14 low–low, 13 low–high, and 9 high–low clusters (Figure 4). The hot spots (high–high clusters) were mainly observed in nine main urban districts, including Bishan, Hechuan, Rongchang, Wulong, Wanzhou, and Chengkou. No hot spots were found in 2005, 2009, 2012, 2017, and 2018.

### 3.4. Spatial Cluster Analysis

**Figure 5:**
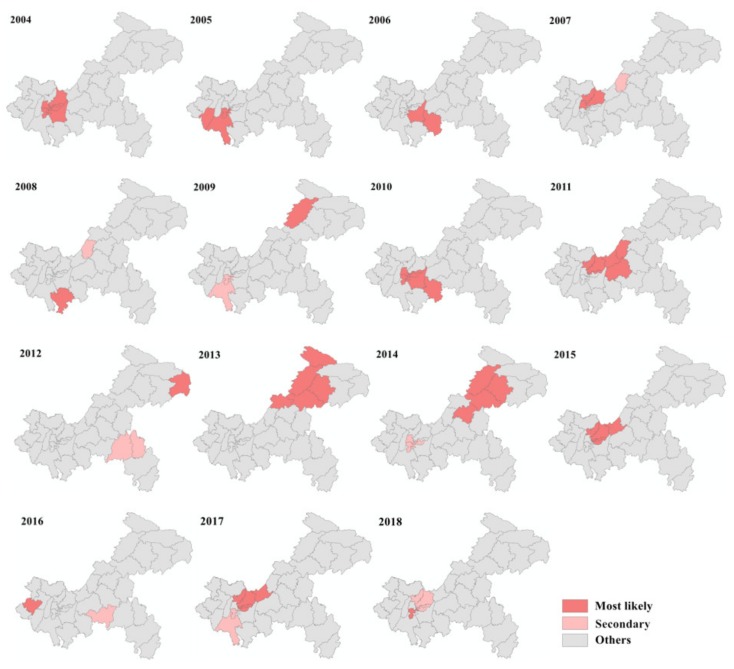
Yearly spatial clusters of mumps in Chongqing from 2004 to 2018.

The purely spatial cluster analysis indicated that the spatial distribution of mumps was not random in Chongqing between 2004 and 2018. The spatial scan identified the 15 most likely clusters and 8 secondary clusters for high-incidence areas of mumps with statistical significance (Table 2). The number of counties included in each cluster ranged from one to eight. The exact locations of clusters varied every year, but they were mainly in the west, midwest, and northeast of Chongqing (Figure 5).

### 3.5. Temporal and Space–Time Cluster Analysis

**Figure 6:**
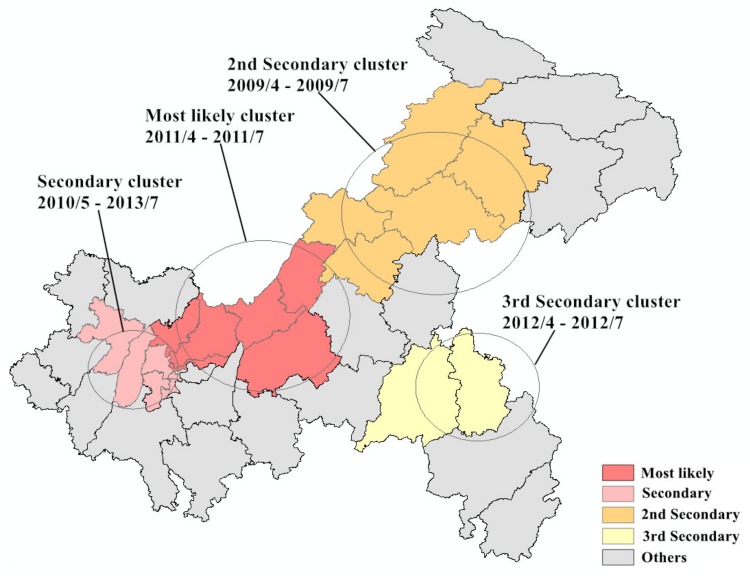
Spatiotemporal clusters of mumps in Chongqing from 2004 to 2018.

The results of the purely temporal scan analysis showed that the time aggregation frame was from March 2009 to July 2013 (*LLR* = 6936.16, *p* < 0.001). Spatiotemporal scan analysis detected four high-incidence clusters of mumps, including 18 counties in Chongqing, from 2004 to 2018. The most likely cluster, covering six counties, was mainly found in the west-central part of Chongqing from April 2011 to July 2011 (*LLR* = 3650.61, *p* < 0.001). The secondary cluster (n = 5), second secondary cluster (n = 5), and third secondary cluster (n = 2) were located in the western, northeastern, and southwestern regions of Chongqing, respectively. The durations of all spatiotemporal clusters were recorded between April 2009 and July 2012 and were included in the temporal cluster detected by the purely temporal scan. The results of the spatiotemporal scan are detailed in Table 3 and mapped in Figure 6.

## 4. Discussion

In this study, we described the epidemiology of mumps in Chongqing in terms of trends and the distribution of high-risk times and groups, and from a geographical point of view, we detected spatial, temporal, and spatiotemporal clusters. During the 15-year study period, the incidence of mumps fluctuated and presented a decreasing trend in general. Although the mumps vaccine was incorporated into the national immunization expansion plan in 2008, the number of mumps cases was not rapidly and considerably brought under control. Several years passed before it showed a downward trend, which was the same as the incidence in the whole country and some other provinces [25,33,34]. This may be related to the accumulation of susceptible children who missed opportunities for vaccination for some reason, the age limit of the vaccinated population, and the periodicity of mumps [35,36,37]. The downward trend of mumps in the last few years indicated that the preventive and control measures were effective. Previous epidemiological studies of mumps in other regions [24,38,39] have shown that the incidence among males is significantly higher than that among females, and the same was true of our findings, which may be due to differential behavioral patterns [40,41]. Males are relatively more active and less hygienic, which increases their exposure to the mumps virus. According to the one-dose MMR vaccination policy in 2008 for children aged 18–24 months, it was estimated that all children under the age of 5 after 2013 would be vaccinated free of charge. Therefore, in 2013–2018, the number of cases in children aged 0–4 years tended to be stable.

Children aged 5–9 years as the high-incidence population had a fluctuating incidence, which might be attributed to the congestion and high density of the school environments in which they live and the weakening of vaccine efficacy over time [36]. Indeed, the prevalence rates of three economically developed municipalities, namely, Beijing, Shanghai, and Tianjin, which introduced the two doses of the routine immunization program, were significantly lower than those of other provinces [33]. The epidemic situation of mumps in this study had a bimodal seasonal distribution, which was roughly consistent with the results of previous studies [24,25,26]. In China, both of these peaks occurred in the student’s school term time, which was linked to the phenomenon that students were the main occupation of mumps patients. Hence, schools and kindergartens should strengthen health management for students and kindergarten children, strictly check their vaccination certificates at admission, and promote health knowledge education.

Studies have confirmed that potential climate factors such as temperature, humidity, and sunshine are significantly correlated with the incidence of mumps in other parts of China [39,42,43,44]. Chongqing, situated in the Chinese southeastern inland, has a different geographical location and climate, so the impact of climate on the disease may be different. Nevertheless, no such study has been carried out in Chongqing, and thus climate can be considered in the future to better predict the prevalence of mumps. Combining the incidence map with the results of global and local spatial autocorrelation analyses showed that the geographical distribution of mumps in Chongqing had spatial heterogeneity, but it was not as significant as that in previous studies [24,25,26]. Little deviation was noted between the locations of high-incidence areas detected by the spatial scan, space–time scan, and LISA map, but these locations were basically consistent. Given that the locations of high-risk clusters changed almost every year, it is necessary to carry out real-time space monitoring. There still existed some high-incidence regions of mumps, mainly in the midwestern, western, northeastern, and southwestern parts of Chongqing, particularly in the nine main urban districts and the counties close to the main urban zone. The temporal cluster determined by the purely temporal scan statistics was consistent with the observed high-prevalence years. In relatively developed urban regions, the higher incidence may be explained by the large population density, large population turnover, and diagnostic level. Some northeastern and southwestern counties, such as Chengkou, Pengshui, and Shizhu, located in mountainous areas with low socioeconomic status and insufficient health care services, also experienced high prevalence in certain years, which may have been caused by the untimely and low coverage of mumps vaccination [45,46]. The actual influencing factors of prevalence in high-incidence counties should be further studied in the future. In addition to the deficiencies of the current immunization policy itself, the two-dose routine immunization program has not been implemented, so the immunization level of the population is at a relatively low level of protection. In the results of the present study, the absence of a global spatial correlation did not mean that significant local clusters were nonexistent, and local autocorrelation analysis can reveal potentially masked local autocorrelation regions [47]. The LISA cluster map has the advantage of intuitively displaying truly local clustering regions. The principles and analytical variables of scan statistics and spatial autocorrelation methods to study the distribution of diseases from the perspective of GIS are different, so some dissimilarities also existed in the high-risk areas detected by each of them. In future studies, we can also consider combining the two methods to better and more completely grasp the geographical features of diseases.

Our findings may help public health agencies develop and improve effective prevention and control strategies and may provide evidence and clues for further research on the related risk factors for mumps. However, this study still has several limitations. First, the data quality of reported mumps cases was related to the monitoring sensitivity and management capacity of each regional system. This surveillance system is a passive information collecting system, which might lead to the underestimation of mumps cases, especially mild cases. Second, some meteorological and socioeconomic factors that may be associated with mumps epidemics were not included in this study for analysis, and so the cause of mumps could not be well explained.

## 5. Conclusions

The incidence of mumps in Chongqing was found to decline and reached a low level in 2018 after several fluctuations, with obvious seasonal variations. Children aged 5–9 years, males, and students were more susceptible to mumps virus. Although this study identified the spatial heterogeneity of mumps and the locations of high-risk areas, the high-incidence clusters varied year by year. Therefore, strengthening real-time spatial early monitoring and warning is required to better control and prevent mumps for targeting local endemic regions, especially at peak periods. Health-related policies should be formulated to take measures to implement intensive and compulsory vaccination, incorporate a second dose of mumps vaccine into routine vaccination procedures for higher and longer-lasting immunity among the young population, and allocate health resources more appropriately.

## Figures and Tables

**Table 1:** Global spatial autocorrelation of mumps in Chongqing from 2004 to 2018.

Year	Moran’s *I*	Z-Score	*p*-Value	Mean	SD
2004	0.4511	4.2581	0.001	−0.0247	0.1117
2005	−0.1398	−1.117	0.134	−0.0227	0.1048
2006	0.0564	0.7658	0.206	−0.0244	0.1055
2007	0.1763	1.979	0.045	−0.029	0.1037
2008	−0.0838	−0.5153	0.315	−0.0301	0.1042
2009	0.0868	1.0407	0.142	−0.0279	0.1102
2010	0.0508	0.6996	0.235	−0.0265	0.1105
2011	0.1251	1.5539	0.063	−0.0337	0.1022
2012	−0.0225	0.049	0.436	−0.0271	0.0945
2013	0.093	1.0947	0.129	−0.0271	0.1096
2014	−0.0498	−0.2262	0.426	−0.0248	0.1108
2015	0.2124	2.4075	0.02	−0.0273	0.0995
2016	−0.0888	−0.6509	0.271	−0.0223	0.1023
2017	0.0376	0.6416	0.252	−0.0328	0.1096
2018	−0.0359	−0.0229	0.464	−0.0335	0.1053

**Table 2:** Yearly spatial clusters of mumps cases in Chongqing from 2004 to 2018.

Year	Cluster Type	Counties (n)	Radius (km)	Observed Cases	Expected Cases	*LLR*	*RR*	*p*-Value
2004	Most likely	8	31.61	5076	1894.39	2381.57	3.90	<0.001
2005	Most likely	4	47.62	2609	1295.83	600.37	2.31	<0.001
2006	Most likely	2	48.38	1083	327.65	587.66	3.77	<0.001
2007	Most likely	3	29.78	1152	367.10	604.56	3.79	<0.001
	Secondary	1	0	350	140.23	115.04	2.61	<0.001
2008	Most likely	1	0	1158	370.77	567.79	3.43	<0.001
	Secondary	1	0	702	262.61	261.85	2.81	<0.001
2009	Most likely	1	0	1760	688.26	622.52	2.77	<0.001
	Secondary	3	47.23	2306	1176.00	471.10	2.14	<0.001
2010	Most likely	8	48.38	4338	2561.94	647.21	1.99	<0.001
2011	Most likely	6	61.20	5575	3130.64	1001.61	2.16	<0.001
2012	Most likely	1	0	1111	229.92	899.31	5.18	<0.001
	Secondary	2	43.74	1123	457.40	360.55	2.59	<0.001
2013	Most likely	5	92.87	4216	2055.66	1092.64	2.54	<0.001
2014	Most likely	4	62.99	2147	1321.45	264.41	1.83	<0.001
	Secondary	5	24.54	1833	1121.74	223.30	1.80	<0.001
2015	Most likely	6	41.12	4513	1878.25	1739.22	3.36	<0.001
2016	Most likely	1	0	658	184.63	378.95	3.82	<0.001
	Secondary	1	0	362	83.80	256.92	4.49	<0.001
2017	Most likely	6	41.12	2599	1612.95	323.31	1.86	<0.001
	Secondary	2	45.44	1402	746.98	254.58	2.04	<0.001
2018	Most likely	1	0	629	261.38	195.58	2.55	<0.001
	Secondary	4	31.61	1466	904.21	175.21	1.80	<0.001

Abbreviations: *RR*—relative risk; *LLR*—log likelihood ratio.

**Table 3:** Spatiotemporal clusters of mumps cases in Chongqing from 2004 to 2018.

Cluster Type	Time Frame	Counties (n)	Radius (km)	Observed Cases	Expected Cases	*LLR*	*RR*	*p*-Value
Most likely	April–July 2011	6	61.20	3819	644.86	3650.61	6.04	<0.001
Secondary	May 2010 to July 2013	5	31.83	9866	4148.18	2936.65	2.47	<0.001
Second Secondary	April–July 2009	5	66.04	3249	689.46	2497.72	4.79	<0.001
Third Secondary	April–July 2012	2	43.74	821	121.36	871.45	6.79	<0.001
